# A recent shift in the *Puccinia striiformis* f. sp. *tritici* population in Serbia coincides with changes in yield losses of commercial winter wheat varieties

**DOI:** 10.3389/fpls.2024.1464454

**Published:** 2024-10-28

**Authors:** Vesna Župunski, Loizos Savva, Diane G. O. Saunders, Radivoje Jevtić

**Affiliations:** ^1^ Laboratory for Phytopathology, Small Grains Department, Institute of Field and Vegetable Crops, Novi Sad, Serbia; ^2^ John Innes Centre, Norwich Research Park, Norwich, United Kingdom

**Keywords:** wheat, wheat yellow rust, wheat yield, yield loss, control

## Abstract

Wheat yellow (stripe) rust, caused by the fungus *Puccinia striiformis* f.sp. *tritici* (*Pst*), is a devastating disease of wheat worldwide. The success of *Pst* is largely due to the pathogens ability to rapidly overcome host resistance, generating new races that are easily dispersed between territories through wind-borne transmission of *Pst* urediniospores. Thus, first signs of entry of new *Pst* races into a region is usually captured by changes in disease severity. To examine any alterations of winter wheat variety response to *Pst* infection in Serbia, we analyzed yield and *Pst* disease severity in field trials conducted in 2014, 2021, and 2023. We specifically focused on analyzing *Pst* disease severity at growth stages related to yield. Associations between qualitative variables (variety, year) and quantitative variables (yield in untreated plots, yield loss, and disease index (DI) of *Pst* infection) were analyzed using Principal Component Analysis with mixed data. A General Linear Model was used to investigate the most influential factors on yield, yield loss, and *Pst* infection. The results indicated that yellow rust disease severity increased over the past decade, suggesting a potential recent change in the *Pst* population in Serbia. Comparative population genetic analysis of *Pst* samples from the 2023 wheat season and those collected in Serbia in 2014 confirmed a potential change in the *Pst* population. In addition, we found that yield losses across wheat varieties varied independently of *Pst* infection levels, indicating that wheat varieties differ in their ability to overcome damage caused by high levels of *Pst* infection. Given that the level of pathogen pressure triggering susceptibility reactions is cultivar-specific, our study highlights the need for a deeper focus on the mechanisms underlying these differences. Expanding our understanding of the interactions between pathogens, plant defense responses, and the ability of cultivars to mitigate yield losses will better equip us to predict and prevent potential yield losses in commercial wheat varieties due to yellow rust in the future.

## 1 Introduction

*Puccinia striiformis* f.sp. *tritici* (*Pst*) is the causal agent of wheat yellow (stripe) rust, that is one of the most devastating diseases of wheat worldwide and is largely dispersed by air currents that transmit asexual urediniospores over long distances (Zadoks, 1961; Hovmøller et al., 2011). Wheat yellow rust is not transmitted through seed although under high infection intensities *Pst* urediniospores can be observed on host plant ears (Chen et al., 2014). Changing climatic conditions drive the continuous breeding of new varieties for increased fitness in response to these rapidly shifting environments. However, new *Pst* races also regularly emerge that can overcome resistance in introduced wheat varieties making the control of yellow rust challenging. Furthermore, recent shifts in the geographic distribution of *Pst*, particularly in North America, have also been linked to temperature-specific adaptation (Lyon and Broders, 2017), reflecting the high adaptability of *Pst* to environmental pressures. Consequently, changes in pathogens populations affected by changes in climatic conditions, selection pressure of resistant varieties and applied pesticides (Ash and Brown, 1990; Pritsch et al., 2000; Ahmad et al., 2010; Ma et al., 2013; Xi et al., 2015; El Jarroudi et al., 2017; Carmona et al., 2019; Chen, 2020) makes yellow rust control challenging (Chen, 2005; Ali et al., 2014; Hovmøller et al., 2016; Bueno-Sancho et al., 2017).

The potential for pathogens to better adapt to changing environmental conditions are also influenced by the type of reproduction, giving high adaptation abilities to pathogens with sexual reproduction stages in their life cycles. *Pst* is heteroecious, needing two hosts to complete its life cycle; it reproduces asexually on wheat and sexually on *Berberis* spp (Jin et al., 2010). *Pst* diversity is centered in the Himalayan region, where sexual reproduction and thus significant genetic variation occurs, in contrast to the predominant clonal population structure found in Europe, America, and Australia (Hovmøller et al., 2011; Ali et al., 2014). While mutation is known to serve as a major source of genetic variation, generating new alleles and genotypes, sexual, parasexual, and somatic recombination also influence genotype diversity (Hovmøller et al., 2011).

Before 2011, the population of *Pst* in Europe was mainly clonal, relying on mutations with minimal influence from sexual recombination (Hovmøller et al., 2002). Consequently, new virulent *Pst* strains arose due to stepwise mutation within these clonal populations (Linde et al., 2002; de Vallavieille-Pope et al., 2012). However, this situation abruptly changed in 2011, with the discovery of two new *Pst* races, termed ‘Warrior’ and ‘Kranich’, that were detected across numerous European countries and had greater genetic variability than previous clonal populations (Hovmøller et al., 2016). These new exotic *Pst* lineages then rapidly replaced the previously known *Pst* population in Europe (Hubbard et al., 2015) and harbored virulence against varieties that were previously resistant to the prevailing *Pst* races, making rust control much more challenging (Sørensen et al., 2014). The Warrior race has also spread to North Africa since its appearance in Europe, challenging wheat production (Ali et al., 2014; Hovmøller et al., 2016; Shahin, 2020). These findings underscore the reality that, despite ongoing efforts to develop wheat varieties resistant to existing *Pst* populations, the emergence of new *Pst* races can trigger outbreaks of epidemic proportions, even on a continental scale (Brown and Hovmøller, 2002).

The first observations of yellow rust in Serbia occurred at Rimski Šančevi in 1997 within a genetic collection comprising 630 genotypes of wheat and wild relatives (Jevtić et al., 1997). The first warning of its heightened threat was issued in 2007 as part of the ADAGIO Project (Adaptation of Agriculture in European Regions at Environmental Risk Under Climate Change) (Jevtić et al., 2009; Mihailovic et al., 2009; Jevtić and Župunski, 2023). This increase in yellow rust infections in 2007 was associated with increased drought and heat likely due to climate change and coincided with expanded geographic distribution of *Pst*. However, until 2014, the related wheat leaf rust pathogen, *Puccinia triticina*, remained the predominant wheat rust species in Serbia. Yet, during the 2013/2014 production season, winter temperatures exceeded average temperatures since 1964 leading to yellow rust predominance over leaf rust and jeopardizing wheat production (Jevtić et al., 2017, 2020). The outbreak of yellow rust in Serbia in 2014 was attributed to influx of the *Pst* Warrior race (Jevtić et al., 2017, 2020), with disease indices (DI) ranging from 40% to 60% (Jevtić et al., 2020).

The studies primarily focus on monitoring shifts in the yellow rust population and its effects on wheat production, but the number of model genotypes used per study to assess the impact of yellow rust on agronomic traits is usually limited to a few well-known cultivars categorized as either susceptible or resistant. However, the variation in responses among different susceptible cultivars to yellow rust infection is often overlooked, as is the combined effect of biotic and abiotic stressors on disease occurrence (White et al., 2011; Juroszek and von Tiedemann, 2013). The lack of information on factors that affect diversity of reactions of susceptible varieties to yellow rust infection, and how they compensate for yield losses makes predictions of yellow rust occurrence more challenging and limits the effectiveness of methods aimed at preventing agronomic losses.

Considering the ability of *Pst* to rapidly overcome host plant resistance, and the complexity of signal cross-talk controlling plant responses to abiotic and biotic environmental factors, we set out to examine the yield response of winter wheat varieties to *Pst* infection. In particular, we decided to assess how different levels of *Pst* infection affect yield responses of commercial winter wheat varieties, and the potential for different host genotypes to compensate for yield losses after delayed fungicide application under high established *Pst* infection levels. Our results, indicate that the level of pathogen pressure that triggers susceptible reactions is cultivar-specific, with diversity in the ability for commercial winter wheat varieties to overcome damage caused by high levels of *Pst* infection. Furthermore, through population genetic analysis we also uncovered a potential recent change in the *Pst* population in Serbia that could have an impact on disease severity in the region.

## 2 Materials and methods

### 2.1 Wheat field trials

Field trials were conducted in 2014, 2021, and 2023 at Rimski šančevi (45°19′47.30″ N, 19°50′44.00″ E, at an altitude of 85.7 m), in Vojvodina, the northern province of Serbia to assess resistance/susceptibility of commercial winter wheat varieties to *Pst.* Trials were undertaken in a slightly carbonated loamy chernozem soil with reduced cultivation practices. A total of 17 and 89 commercial varieties in 2014 and 2023 were assessed, respectively. To track the yield potential of commercial varieties in the absence of *Pst* infection, data from 2021 were also considered in the study, since no yellow rust was reported in 2021 due to unfavorable climatic conditions.

Field trials were conducted using methodology recommended by CIMMYT (1979). Accordingly, each genotype was sown in one un-replicated 5 meter row or using smaller row lengths but with replicates so that the sum of all row lengths was equal to 5 meters. To enable assessment of yield in the presence and absence of yellow rust, varieties were sown in plots of 5 metre^2^ and divided into fungicide-untreated area (4 m^2^) and fungicide-treated area (1 m^2^). Fungicide treatments were applied at recommended dosage rates at growth stage BBCH 65. The active ingredients used were: 100 g/L (10.3% w/w) prothioconazole, 100 g/L (10.5% w/w) tebuconazole and 250 g/L (26.3% w/w) spiroxamine (2014) and 75 g/l benzovindiflupyr 150 g/l, prothioconazole (2023). Fungicides were applied using calibrated field crop sprayers with fan nozzles, at 300 kPa pressure, and 200 L of water per hectare. The mean sowing date for winter wheat was October 20 (optimal time of sowing), and the mean harvest date was June 30.

### 2.2 Disease severity assessments

Disease severity of obligate pathogens were made at the growth stage known to be highly related to yield (BBCH 71-73, kernel watery; early milk) (Wegulo et al., 2009). A modified Cobb’s scale was used for disease severity assessments (Peterson et al., 1948; Corazza and Islongo, 1987). Since yellow rust predominated over the rest of the obligate pathogens in 2014 and 2023, the disease indices (%) of yellow rust were calculated by taking into account disease incidence and average disease severity (Cao et al., 2014).

### 2.3 Wheat yield assessment

Wheat yield per cultivar was measured separately for each fungicide-treated and fungicide-untreated plot at 15% water content. Yield loss (%) was determined as the average reduction in yield in fungicide-untreated plots compared to the yield in fungicide-treated plots (Equation 1). Yield gain (%) was calculated by considering average yield in fungicide-untreated plots and average yield achievement in fungicide-treated plots (Equation 2).


(1) Y(%)=((Y1-Y2)/Y1)×100

(2) Y(%)=((Y1-Y2)/Y2)×100

Y1—Average grain yield in fungicide-treated plots.

Y2—Average grain yield in fungicide-untreated plots

### 2.4 *Pst* population genetic analysis

A total of 26 *Pst*-infected wheat samples were collected from the field trials in 2022-2023 and were subjected to RNA extraction using a Qiagen RNeasy Mini Kit (Qiagen, UK) (Hubbard et al., 2015; Boshoff et al., 2020) (**Supplementary Table S1**). Extracted total RNA were analyzed for quality and quantity using a Qubit fluorometer (Thermo Fisher Scientific, USA). Subsequently, cDNA libraries were prepared using an Illumina TruSeq RNA Sample Preparation Kit (Illumina, USA) and sequenced on an Illumina NovaSeq instrument by Azenta Life Sciences (USA). In addition, genomic or RNA-seq datasets for a total of 169 *Pst* isolates collected across 27 countries were obtained from public repositories (Hubbard et al., 2015; Boshoff et al., 2020) (**Supplementary Table S1**). Reads were aligned for each sample against the *Pst* reference genome (*Pst* isolate Pst104e; (Schwessinger et al., 2018) using the Burrows-Wheeler Alignment (BWA) tool, version 0.7.5 (Li and Durbin, 2009) for genomic reads, and the Star alignment tool, version 2.5 (Dobin et al., 2013) for RNA-seq reads. SAMtools version 0.1.19 (Li et al., 2009) was used to generate pileups and identify single nucleotide polymorphisms (SNPs). This information was utilized to create the consensus sequences for each gene per *Pst* isolate as described previously (Hubbard et al., 2015) Phylogenetic analysis was conducted using 4,682 gene models with coverage in at least 80% of the 195 *Pst* isolates using FastTree version 2.1 and default settings allowing inference of maximum-likelihood (Price et al., 2010).

### 2.5 Statistical analyses

Since principal component analysis (PCA) requires quantitative and multiple correspondence analysis (MCA) qualitative variables only, associations between qualitative variables (variety, year) and quantitative variables (yield in untreated plots, yield loss, and DI of yellow rust) were analyzed using PCA with mixed data (PCAmix). A general linear model (GLM) was used to investigate the most influencing factors on yield, yield loss, and *Pst* infection of commercial winter wheat varieties in 2014 and 2023. In GLM analysis, predictor variables for evaluating the influencing factors on yield in untreated plots and yield losses were year, variety, and their interaction, while *Pst* infection was considered as a covariate variable. The relationship between yield and yield losses with *Pst* infection in 2014 and 2023 was analyzed using Spearman’s coefficient of correlation. Tukey pairwise comparison was used to identify whether yield achievements in treated and untreated plots differed among growing seasons. Monthly averages of temperature, total rainfall, and humidity during the 2014, 2021, and 2023 growing seasons were recorded for the experimental site using the weather station of the Republic Hydrometeorological Institute of Serbia located at Rimski šančevi (RHMZ) (http://www.hidmet.gov.rs/). All statistical analysis was performed using Minitab 17 Statistical Software and XLSTAT in Microsoft Excel. The ‘ggplot2’ package in R software was used for the visualization of PCAmix analysis.

### 2.6 Data availability

Newly generated RNA-seq data was deposited in the European Nucleotide Archive (ENA) database under the accession number: PRJEB77002. Accession numbers for additional genomic and RNA-seq data used in the phylogenetic analysis is provided in **Supplementary Table S1**.

## 3 Results

### 3.1 Yellow rust severity increased over the past decade in Serbia

**Figure 1:**
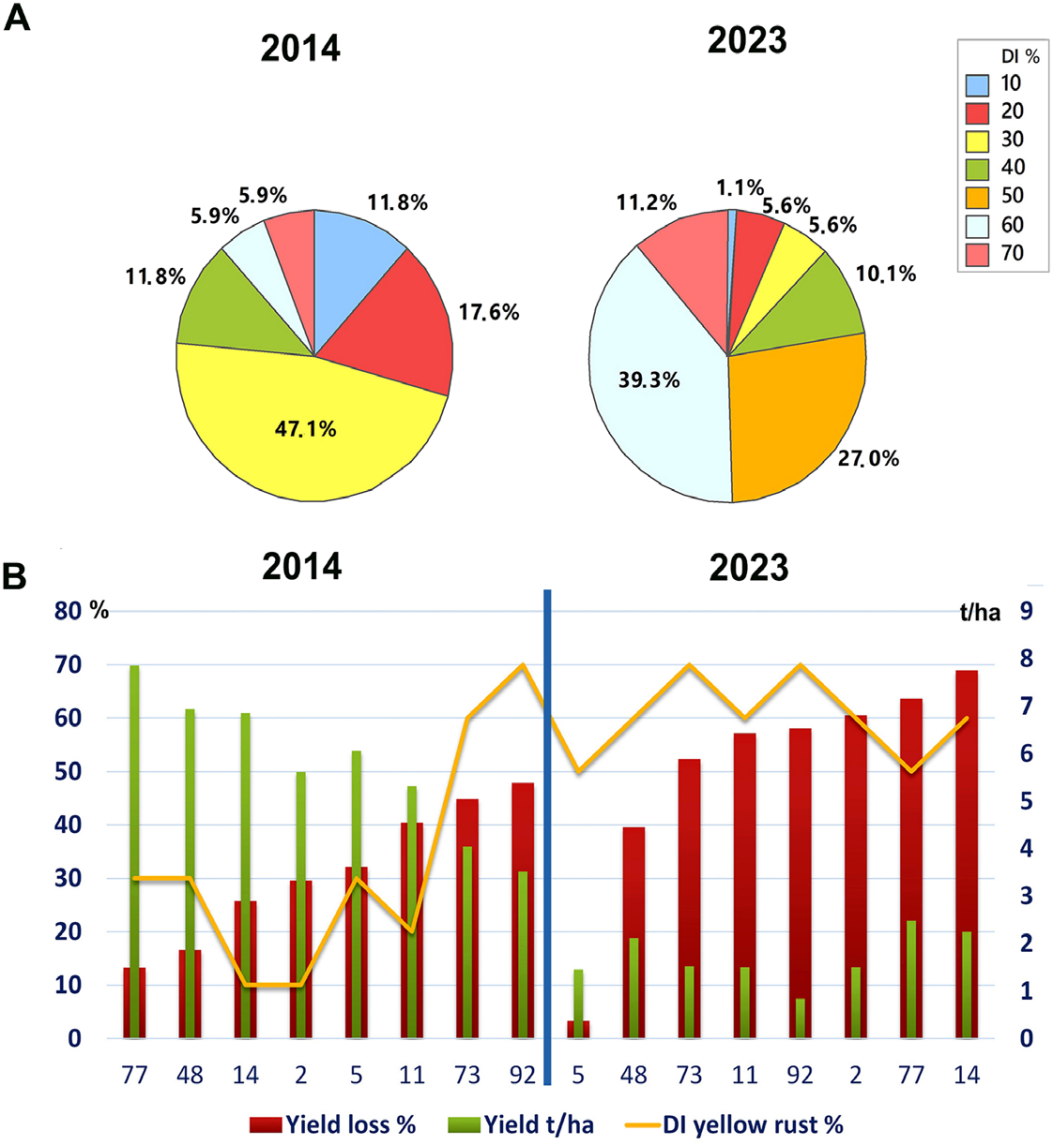
Yellow rust severity was much higher in the 2023 than the 2014 growth season. **(A)** The level of yellow rust disease incidence (DI) ranged from 10% to 70% in 2014 and 2023 across commercial wheat varieties grown in untreated plots at Rimski šančevi locality. A total of 17 and 89 commercial varieties in 2014 and 2023 were assessed for yellow rust severity at the growth stage known to be highly related to yield (BBCH 71-73, kernel watery; early milk). Disease indices (%) were calculated by taking into account yellow rust disease incidence and average disease severity. Significant increase in Pst infections, with an average severity of 31.2% for 17 cultivars analyzed in 2014 and 51.9% across the 89 cultivars analyzed in 2023 was detected. **(B)** Six out of eight cultivars, which had a moderate level of resistance to *Pst* in 2014, was highly susceptible in 2023, with reduced yield. This indicates that the aggressiveness of Pst races may have changed between 2014 and 2023. The yellow rust disease index, yield, and yield losses of 8 commercial winter wheat varieties were assessed in both 2014 and 2023 at the Rimski Šančevi locality. t/ha, tons per hectare.

To assess any changes in yellow rust disease severity since 2014 in Serbia, we analyzed the average disease indexes (DI) in untreated plots in the 2014 and 2023 seasons. We found a significant increase in *Pst* infections, with an average severity of 31.2% for 17 cultivars analyzed in 2014 and 51.9% across the 89 cultivars analyzed in 2023 (p< 0.001). Although both 2014 and 2023 were favorable for *Pst* infection, we noted greater aggressiveness of *Pst* in untreated plots in 2023 (**Figure 1A**). Accordingly, in 2014, the majority of wheat genotypes (47.1%) were infected with a DI of 30%, and only 11.8% of genotypes were infected with yellow rust at 60% and above. Whereas in 2023, 50.5% of genotypes were infected with a DI of 60% and above (**Figure 1A**).

Furthermore, comparison of 8 wheat genotypes cultivated in both 2014 and 2023 revealed that 6 out of 8 had lower *Pst* infection in 2014 than in 2023. Only 2 out of 8 of these common varieties had a consistent level of susceptibility in both years, with DI’s exceeding 60%. In addition, yields in untreated plots of these 2 varieties in 2014 reached 3.5 and 4 t/ha, but were below 1.5 t/ha in 2023 (**Figure 1B**). Tukey pairwise comparison showed that yields in untreated plots of the 8 varieties were significantly higher in 2014 (5.8 t/ha) than in 2023 (1.5 t/ha) (p< 0.001). This indicates that the aggressiveness of *Pst* races may have changed between 2014 and 2023, leading to an increase in DI in varieties previously shown to possess a moderate level of resistance to *Pst.*

### 3.2 Yield losses across wheat varieties varied independent of *Pst* infection levels

**Figure 2:**
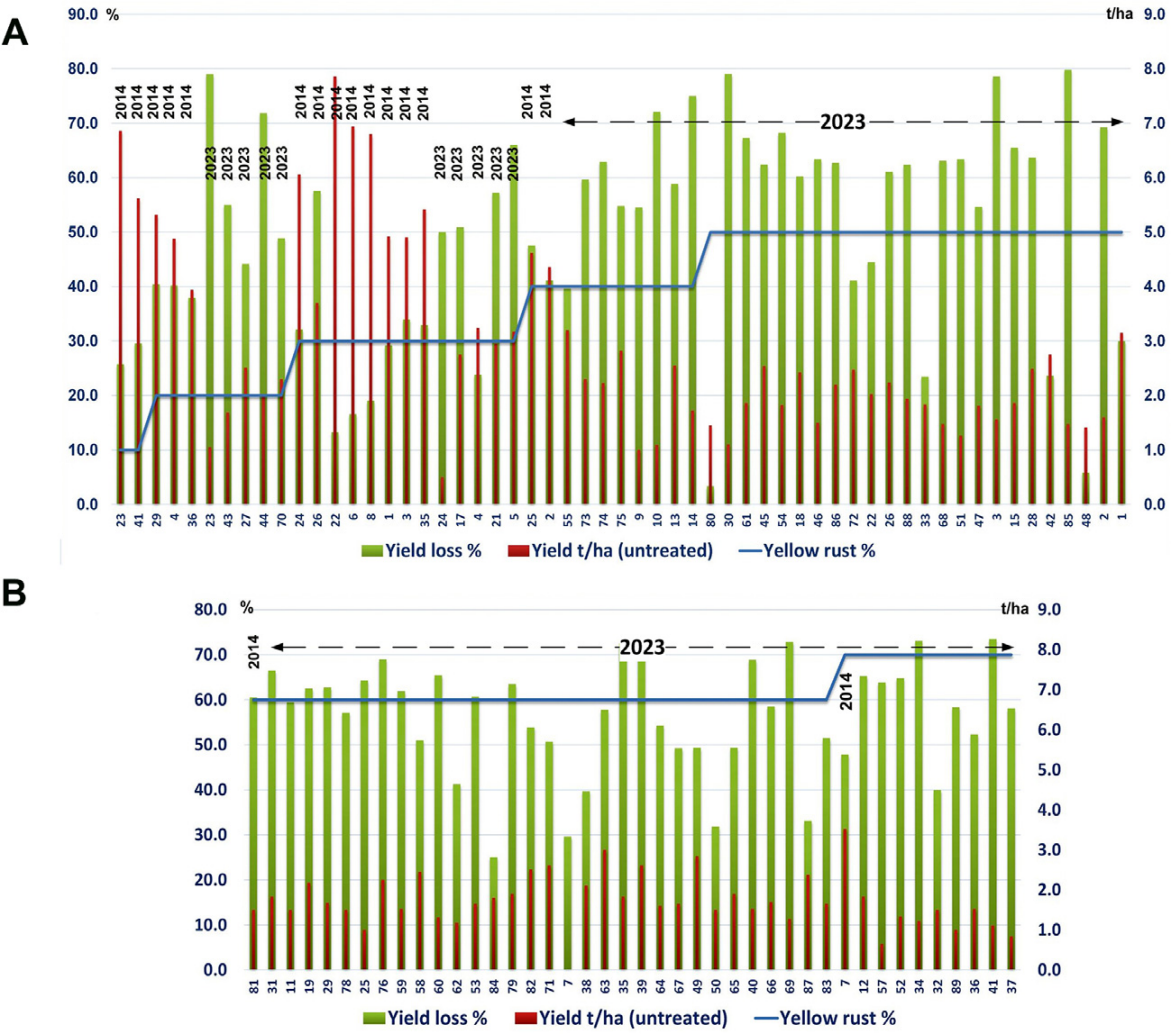
Yield losses across wheat varieties varied regardless of *Pst* infection levels. The yellow rust disease index, yield, and yield losses were assessed for 17 or 89 commercial winter wheat varieties in 2014 and 2023 respectively at the Rimski Šančevi locality. **(A)** Yield loss varied widely with varieties that had a low level of yellow rust infection (20%) in 2014, differing in yield loss in untreated plots from below 40% or well above 50%. Similarly, in untreated plots in 2023, where *Pst* infection levels exceeded 50%, yield losses also varied between genotypes, and ranged from 3% to 80%. **(B)** In untreated plots in 2023, where *Pst* infection levels exceeded 60%, yield losses also varied between genotypes, and ranged from 25% to 75%. Variability in yield losses under the same level of yellow rust infection indicate that for every variety, a specific level of yellow rust infection may need to be reached to trigger yield decrement. t/ha, tons per hectare.

Across wheat varieties with similar levels of *Pst* infection we also noted a diversity in yield losses in both the 2014 and 2023 seasons. For instance, under a low level of yellow rust infection (20%), yields in untreated plots ranged from less than 3 t/ha (yield loss exceeding 50%) to over 4.5 t/ha (yield loss below 40%) (**Figure 2A**). Where high levels of *Pst* infection were recorded (exceeding 50%) we also recorded a variability in yield losses between genotypes, ranging from 3% to 80% in the same year (2023) (**Figures 2A, B**).

**Figure 3:**
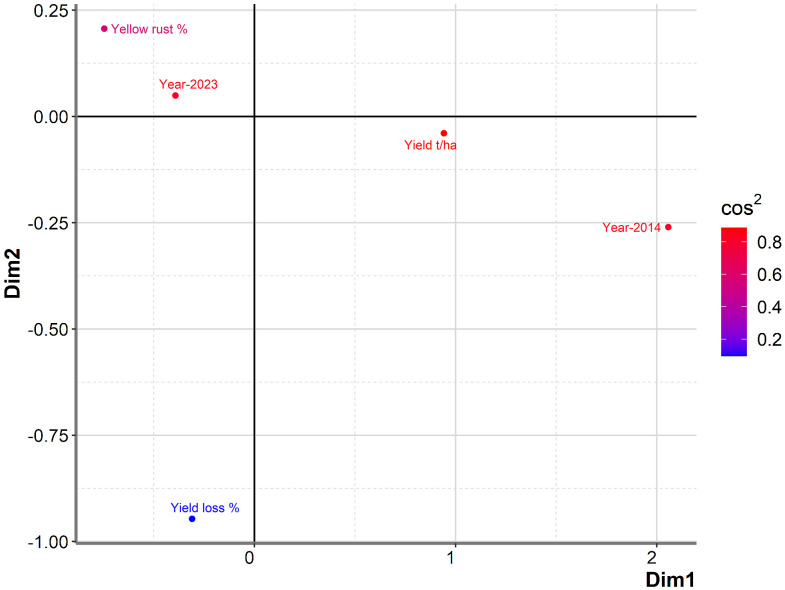
PCAmix analysis showed no notable association between yield and yield losses among commercial wheat varieties assessed in 2014 and 2023. A total of 17 or 89 commercial winter wheat varieties were assessed for yield in 2014 and 2023, respectively, at the Rimski Šančevi locality. Graphical representation of PCAmix analysis between yield, yield loss, disease index of yellow rust and years (2014 and 2023). Dim, dimension; t/ha, tons per hectare.

To further examine any particular associations between yield in untreated plots, yield losses, *Pst* infection, and year, we conducted PCA mix analysis (**Figure 3**). Overall, data from the two years (2014 and 2023) were dispersed in the PCA analysis, with no notable association between yield and yield losses, indicating that they were affected by different factors. However, severity of *Pst* infection was positioned more closely to yield in untreated plots than to yield loss, indicating a potential relationship between yield loss and *Pst* infection. Further analysis of the Spearman coefficient of correlation indicated a significant low correlation between yield loss and *Pst* infection when considering both 2014 and 2023 (r = 0.227, p = 0.019). In contrast, *Pst* infection and yield in untreated plots had a negative moderate significant correlation (r = -0.510, p< 0.001). The squared cosine indicated that year, yield in untreated plots, and *Pst* infection severity had the highest degree of association with the first dimension (red color), while yield loss had a lower association with the first two dimensions (blue color) (**Figure 3**).

**Figure 4:**
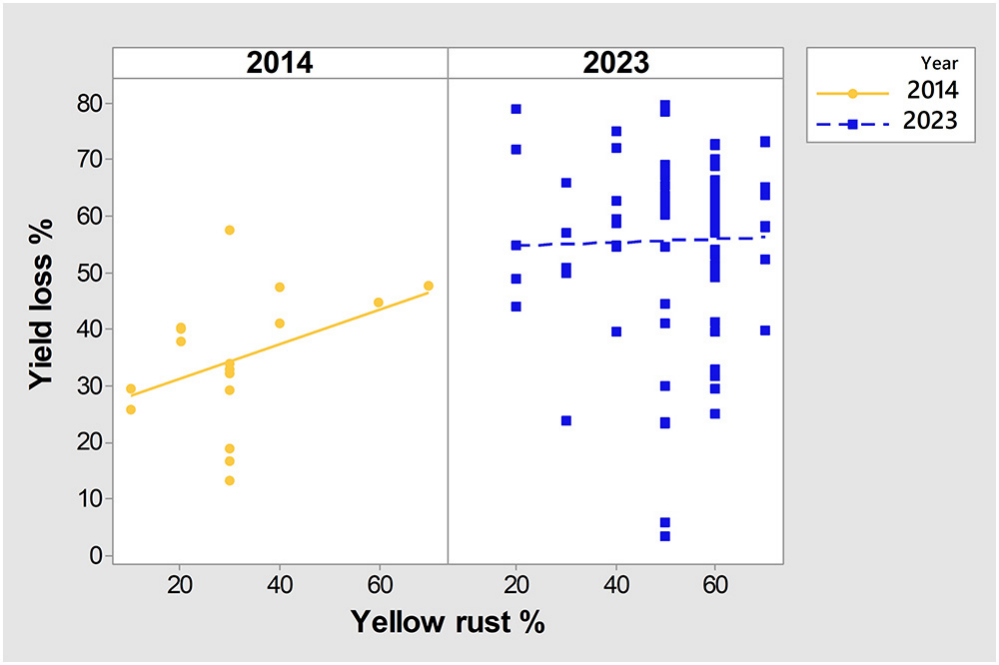
Variation in yield loss for varieties with similar *Pst* disease severity occurred within the same season. A total of 17 or 89 commercial winter wheat varieties were assessed for yield in the 2014 and 2023 seasons, respectively.

We also noted variation in yield loss for varieties with similar *Pst* disease severity within the same season. For instance, the yield loss of 6 varieties infected with yellow rust below 30% in 2014 ranged from 17% to 40%. Moreover, in 2014, 2 varieties (14 and 2) infected with a low level of yellow rust (DI = 10%) showed higher yield losses (26% and 30%) than 2 varieties (77 and 48) infected with a DI of 30%, with yield losses of 13% and 17% (**Figure 1B**). However, variability in yield losses under the same level of yellow rust infection was even more prominent in 2023 when 77.5% of genotypes were infected with a DI exceeding 50%, and when yield losses of those varieties ranged from 3% to 80% (**Figures 2**, **4**). These results indicate that for every variety, a specific level of yellow rust infection may need to be reached to trigger yield decrement.

### 3.3 Fungicide application in 2023 failed to restore yield to levels achieved in the absence of *Pst* infections

**Figure 5:**
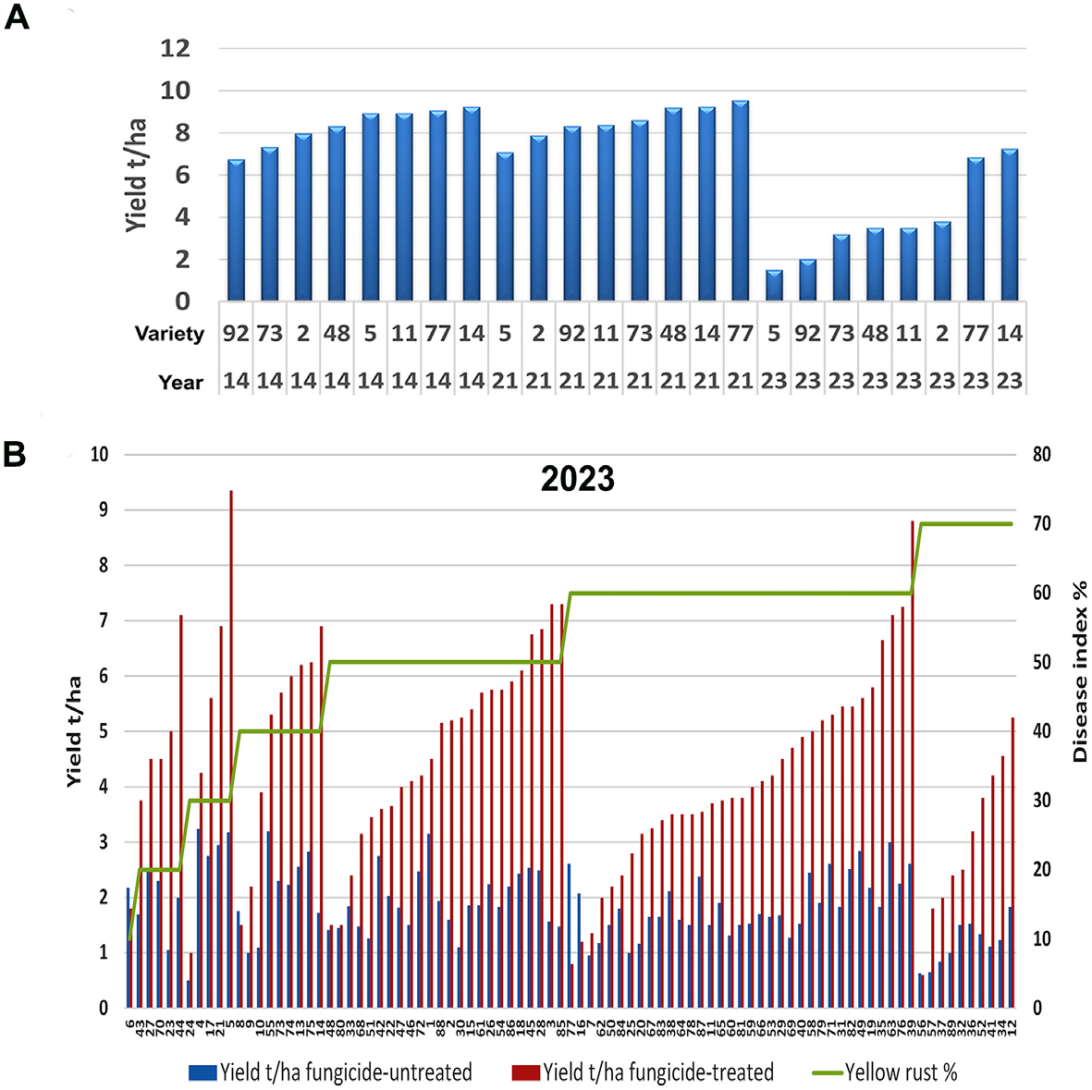
Fungicide application in 2023 did not succeed in restoring yield to levels achieved in the absence of *Pst* infections, and cultivars had different potentials in overcoming damage under high levels of Pst infection. **(A)** Contrary to 2023, delayed fungicide application in 2014 restored yield levels to those of untreated plots in 2021 when there was no yellow rust infection at the Rimski Šančevi locality. **(B)** Yellow rust disease index, yield in fungicide-treated and fungicide un-treated plots were tested in 2023 at Rimski šančevi locality on 89 commercial winter wheat varieties and after delayed fungicide treatment, yields ranged from 1 t/ha to nearly 9 t/ha, even under the same yellow rust infection level exceeding 50%. t/ha, tons per hectare.

We noted that average yield in fungicide-treated plots of the 8 cultivars tested in both 2014 and 2023 was higher in 2014 (8 t/ha) than in 2023 (4 t/ha). To determine if this could be linked to climatic factors, we assessed monthly temperature and precipitation levels in 2014, 2021, and 2023. We included 2021 in our analysis as *Pst* infections were generally absent in this year. In general, we found that yield levels in 2014, 2021 and 2023 could not be linked to differences in temperature and precipitation between years (**Table 1**). For instance, in the absence of *Pst* infections in 2021, we found that wheat varieties exhibited similar yields to the fungicide-treated plots assessed in 2014 (**Figure 5A**). Only 2 out of 8 varieties cultivated in both 2014 and 2023 exhibited yields higher than 6 t/ha after delayed fungicide treatment in 2023. Thus, the lower yield in fungicide-treated plots in 2023 could not be attributed to any specific climatic factors analyzed.

**Table 1:** Temperature (T.) and precipitation (Prec.) data from January to May in 2014, 2021, and 2023 at Rimski šančevi locality.

	T. Jan	Prec. Jan	T. Feb	Prec. Feb	T. Mar	Prec. Mar	T. Apr	Prec. Apr	T. May	Prec. May
2014	4.2	24.3	6.1	9.2	9.9	49.5	13.1	51.2	16.3	202.1
2021	3.3	44.7	5.1	59.5	6.2	42.8	9.6	55.1	16	62.9
2023	4.9	66.4	3.6	57.2	9	25.3	10.4	63.9	17.2	124.8
Aver. 2006-2023	1.4	43.5	3.3	44.1	7.4	43	12.8	43.5	17.3	91

We also noted diversity in genotype potentials in overcoming damage occurring under high levels of *Pst* infection resulted in diversity in yield achievements in fungicide-treated plots. For instance, in yield analysis of 89 cultivars in fungicide-treated plots in 2023, yields in untreated plots did not exceed 3 t/ha, but yields after delayed fungicide treatment ranged from 1 t/ha to almost 9 t/ha under the same level of yellow rust infection that exceeded 50% (**Figure 5B**). These results indicate that commercial varieties did not respond in the same manner to fungicide application in 2014 and 2023.

### 3.4 Potential recent change in the *Pst* population in Serbia

**Figure 6:**
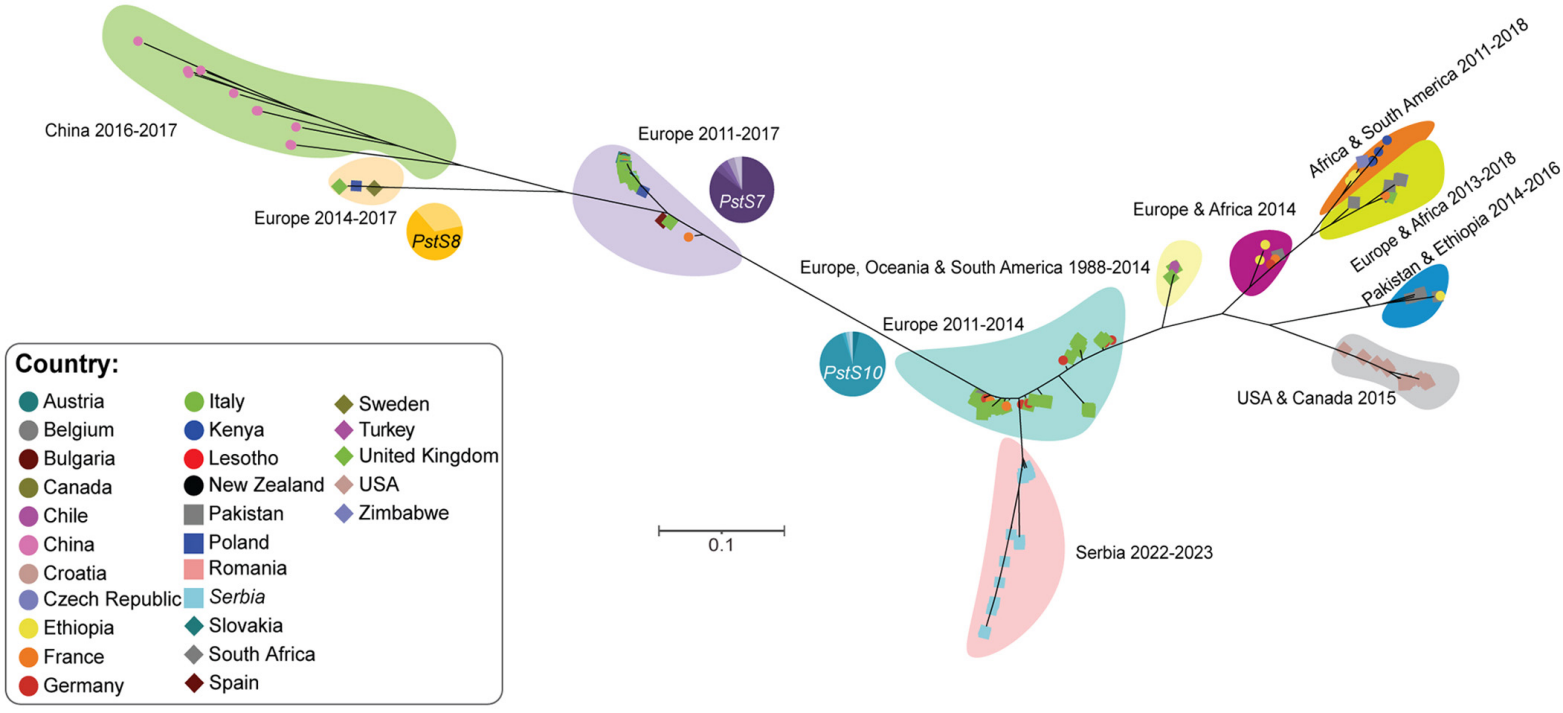
Phylogenetic analysis revealed that *Pst* isolates collected in 2022/23 in Serbia form a distinct clade. A total of 195 genomic and transcriptomic datasets representing *Pst* isolates from 27 countries were used for phylogenetic analysis. The third codon position of 4,682 gene models was extracted for each *Pst* isolate and phylogenetic analysis performed using a maximum likelihood approach. The proportion of *Pst* isolates belonging to three known race groups (PstS7, PstS8 and PstS10) are indicated with pie-charts next to the corresponding shaded area of the phylogeny.

Due to changes in yellow rust disease severity noted in Serbia between 2014 and 2023, we decided to analyze the *Pst* population composition. We collected 26 *Pst*-infected wheat samples from the 2022-23 wheat season and subjected these samples to transcriptome sequencing. To determine the relationship between *Pst* isolates from Serbia in 2022/23, those prevalent in Serbia in 2014 and the broader dominant global *Pst* race groups, we gathered publicly available genomic and transcriptomic data from an additional 169 *Pst* isolates collected across 27 countries and conducted population genetic analysis (**Supplementary Table S1**). Phylogenetic analysis of these 195 *Pst* isolates indicated that *Pst* isolates identified in Serbia in 2022/23 formed a distinct clade that branched from the PstS10 race group (**Figure 6**). Whereas *Pst* isolates collected in Serbia in 2014 clustered within a clade that contained *Pst* isolates predominantly assigned to the PstS7 race group. This analysis supports a potential recent diversification within the *Pst* population detected in Serbia that coincides with the increase in yellow rust disease severity in the region.

## 4 Discussion

In this study, we demonstrated that yield achievements of susceptible wheat genotypes are not uniform in years favorable for *Pst* infection. It appears that for every wheat variety, there exists a specific level of yellow rust infection that triggers yield decrement. As little was known about the potential of fungicides to reduce yield decrement if applied later in the growing season when yellow rust disease severity index (DI) exceeds 20%, we analyzed fungicide effects on yield responses of susceptible genotypes. These results indicate that this relationship is complex and highly associated with the aggressiveness of the dominant *Pst* race(s) and the capabilities of the host plant to overcome damage caused by high levels of *Pst* infection. This indicates that more research is needed to elucidate the factors affecting plant responses to high levels of *Pst* infection and the potential of fungicide treatments to reduce yield losses under these circumstances.

Beyond the presence of the pathogen, climatic conditions are an important factor influencing pathogen outbreaks, alongside the susceptibility of host plants. It is known that elevated temperatures in January and February favor *Pst* infection in Serbia’s agro-ecological conditions. In Serbia, from 2014 to 2023, temperatures in January varied by almost 10°C, and in February by 7°C. Total precipitation also ranged from 1 to 65.5 mm in March and 11.1 to 74.5 mm in April. In 2014, yellow rust predominated over leaf rust for the first time in Serbia, causing enormous damage in wheat production areas (Jevtić et al., 2017, 2020), with the ‘Warrior’ race of *Pst* causing the epidemic in Serbia in 2014 (Jevtić et al., 2017, 2020). Whereas, in 2023 we detected a diversification in the *Pst* isolates identified, with all samples analyzed clustering in a clade branching from the PstS10 race group. This indicates a potential shift in the dominant *Pst* race present in Serbia in the past decade.

Virulence phenotypes of yellow rust are categorized into genetic lineages and designated with “Pst” followed by a sequential number (Ali et al., 2014; Hovmøller et al., 2016; Thach et al., 2016). Yellow rust races in the Northwestern European population, identified before 2011, belonged to a single clonal lineage known as PstS0 (Ali et al., 2014). From 2011 onwards, yellow rust races from the PstS7 (Warrior), PstS8 (Kranich), and PstS10 lineages became widespread in Europe, accounting for over 80% of the examined isolates (Ali et al., 2014; Hubbard et al., 2015; Hovmøller et al., 2016). After its appearance in Europe, PstS7 also emerged in North Africa (Ali et al., 2014; Hovmøller et al., 2016; Shahin, 2020). PstS10, previously referred to as Warrior (-), has been the dominant lineage in most parts of Europe since 2014 (Ali et al., 2014). Virulence pathotypes that predominates in other parts of the world are: PstS, closely related to PstS2, predominates in North America while PstS2 in East Africa (Hovmøller et al., 2008; Ali et al., 2014; Walter et al., 2016); PstS3 is widespread across West Asia, southern Europe, and North Africa (Ali et al., 2014); PstS4 comprises races primarily found on triticale in Northern Europe (Hovmøller et al., 2008; Hovmøller et al., 2016); Two races with distinct microsatellite profiles fall under PstS5 (Ali et al., 2014; Hovmøller et al., 2016; Thach et al., 2016) and PstS6 is another lineage common in East Africa (Ali et al., 2014; Hovmøller et al., 2016; Thach et al., 2016).

The occurrence of a more aggressive *Pst* race in Serbia in 2023 was also associated with lower yields in untreated plots compared to 2014. Additionally, it has been reported that varietal resistance to yellow rust prevents yield losses, and that yield responses of single susceptible varieties could differ in years conducive to yellow rust (Zhou et al., 2022). The study by Jevtić et al. (2017) yielded similar results, where the diversity in yield loss responses of susceptible cultivars to leaf rust and powdery mildew ranged from 20% to 50% over a ten-year period, despite the same level of leaf rust and powdery mildew infection at 20%. However, our study found that there might be a specific level of yellow rust infection that triggers yield decrement individually for each variety, and that susceptible varieties, although infected with high levels of yellow rust, can respond differently to the damage caused. This is consistent with similar analysis conducted for Fusarium head blight (FHB), where each wheat genotype was shown to tolerate a specific range of FHB disease pressure without showing a significant difference in expression of yield potential. Furthermore, it has been shown that defense mechanisms towards FHB are genotype-specific, indicating that resistant genotypes exhibit unique transcriptome profiles during *Fusarium*-host interactions (Pan et al., 2018).

Although wheat pathogens evolve in response to agro-ecological conditions, the combined effect of biotic and abiotic stressors on disease occurrence has frequently been overlooked in research (White et al., 2011; Juroszek and von Tiedemann, 2013). Heeb et al. (2019) advocated for a climate-smart pest management (CSPM) approach, yet they acknowledged the formidable challenge of creating a comprehensive model to predict short-term climate change-induced pest outbreaks at the local level. Understanding that the regulatory network of plant responses to abiotic and biotic stressors and their effects on yield consist of many components that may function antagonistically or synergistically (Glazebrook, 2005; Yasuda et al., 2008; Kissoudis et al., 2014). Our study highlights that when assessing yield achievements and cultivar responses to different levels of yellow rust pressure, more attention should be given to the combined effects of abiotic and biotic stressors. Furthermore, although the majority of studies have addressed fungicide efficacy in lowering host plant infection under diverse levels of pathogen pressure and have also investigated the most efficient timing for fungicide application, little is known about host plant responses to fungicide treatments when disease development is aborted later in the growing season and how it affects final yield achievements.

We found that yield loss between different genotypes in untreated plots ranged from 3% to 80% under yellow rust infection of 50%. This indicates that fungicide efficacy in reducing yield losses, if applied later in the growing season on susceptible genotypes, is complex. Generally, it can be expected that yield losses would be greater if susceptible varieties are infected in the early growth stages (Ash and Brown, 1990; Gaunt and Cole, 1991; Murray et al., 1995), due to the complexity of effects of *Pst* infection on all yield parameters (Ash and Brown, 1990). In our study, the first visible symptoms were recorded at the 30-32 BBCH (Stem elongation) in 2014, and at 20-23 BBCH (Tillering) in 2023. The higher aggressiveness of *Pst* races and much earlier infection in 2023 could have led to the lower yield achievements in untreated plots in 2023 compared to 2014. However, our study also indicated a significant difference in varietal responses to delayed fungicide application when high levels of *Pst* infections were already established. Although fungicide treatment resulted in the abortion of yellow rust development in later growth stages, varieties in our study showed different potential to recover from the damage caused by *Pst* infection and reimburse yield distortion. Consequently, our study indicates that more research should be done to elucidate mechanisms affecting host plant recovery from high levels of *Pst* infection, which would directly influence studies dealing with fungicide efficacy in preventing yield losses, especially when a high level of yellow rust infection is reached.

Establishing correlations between genotype and phenotype is essential for breeding wheat that is better adapted to the changing climate. This challenge is compounded by the fact that plant growth in natural habitats is influenced by a combination of abiotic and biotic stressors. Most existing research has concentrated on plant responses to individual stressors, leading to a limited understanding of adaptation to multiple stress factors. Given that 60% of expression changes under combinatorial stress conditions could not be predicted based on responses to individual stressors (Kissoudis et al., 2014), it can be inferred that the differences between susceptible and resistant plant reactions may be more closely linked to variations in the timing and magnitude of responses to combined stressors, rather than solely to the expression of individual genes (van Loon et al., 2006). There are documented instances of disease resistance being compromised by elevated temperatures and humidity, evidence suggests that temperature variations—rather than fixed thresholds—affect resistance mechanisms against yellow rust in wheat (Bryant et al., 2014). Additionally, drought and salinity have been shown to impact pathogen resistance responses, and the interaction between plants and pathogens can further influence plant responses to abiotic stress (Kissoudis et al., 2014). As a result, analyzing the association between morphological and physiological resistance traits conferred by adult plant resistance (APR) genes against obligate pathogens, alongside abiotic stressors and the expression of abiotic stress-related genes under field conditions, could yield a more detailed genotype profile. This, in turn, would aid in making informed decisions for breeding programs aimed at improving resistance to both biotic and abiotic stressors.

Given that phenotyping in plant breeding will always be an important practice, as determination of genetic variability may not always explain the complexity of phenotype markers (Velu and Singh, 2013), this study filled relevant knowledge gaps to facilitate the improvement of screening methods for winter wheat varieties’ responses to yellow rust infection. Our results indicated that the capabilities of commercial winter wheat varieties to overcome damage caused by high levels of *Pst* infection (>50%) are different, which affects fungicide efficacy in preventing yield losses. Our findings also suggest that the level of pathogen pressure that triggers susceptibility reactions of varieties is cultivar-specific and needs to be investigated in more detail in order to ensure more reliable yellow rust risk prediction in the future.

## Data Availability

Newly generated RNA-seq data was deposited in the European Nucleotide Archive (ENA) database under the accession number: PRJEB77002. Accession numbers for additional genomic and RNA-seq data used in the phylogenetic analysis is provided in **Supplementary Table S1**.

## Supplementary material

Supplementary Table S1 Details of the *Pst* isolates used for conducting the phylogenetic analysis.
